# Induction of SerpinB2 and Th1/Th2 Modulation by SerpinB2 during Lentiviral Infections *In Vivo*

**DOI:** 10.1371/journal.pone.0057343

**Published:** 2013-02-27

**Authors:** Lee D. Major, Thomas S. Partridge, Joy Gardner, Stephen J. Kent, Robert de Rose, Andreas Suhrbier, Wayne A. Schroder

**Affiliations:** 1 Department of Immunology, Queensland Institute of Medical Research, Brisbane, Queensland, Australia; 2 School of Chemistry and Molecular Biosciences, University of Queensland, Brisbane, Queensland, Australia; 3 Department of Microbiology and Immunology, University of Melbourne, Victoria, Australia; 4 School of Biomolecular and Physical Sciences, Griffith University, Nathan, Queensland, Australia; South Texas Veterans Health Care System and University Health Science Center San Antonio, United States of America

## Abstract

SerpinB2, also known as plasminogen activator inhibitor type 2, is a major product of activated monocytes/macrophages and is often strongly induced during infection and inflammation; however, its physiological function remains somewhat elusive. Herein we show that SerpinB2 is induced in peripheral blood mononuclear cells following infection of pigtail macaques with CCR5-utilizing (macrophage-tropic) SIV_mac239_, but not the rapidly pathogenic CXCR4-utilizing (T cell-tropic) SHIV_mn229_. To investigate the role of SerpinB2 in lentiviral infections, SerpinB2^−/−^ mice were infected with EcoHIV, a chimeric HIV in which HIV gp120 has been replaced with gp80 from ecotropic murine leukemia virus. EcoHIV infected SerpinB2^−/−^ mice produced significantly lower anti-*gag* IgG1 antibody titres than infected SerpinB2^+/+^ mice, and showed slightly delayed clearance of EcoHIV. Analyses of published microarray studies showed significantly higher levels of SerpinB2 mRNA in monocytes from HIV-1 infected patients when compared with uninfected controls, as well as a significant negative correlation between SerpinB2 and T-bet mRNA levels in peripheral blood mononuclear cells. These data illustrate that SerpinB2 can be induced by lentiviral infection *in vivo* and support the emerging notion that a physiological role of SerpinB2 is modulation of Th1/Th2 responses.

## Introduction

SerpinB2 is a member of the clade B or ovalbumin-like serine protease inhibitor (ov-serpin) subgroup of the serpin superfamily. SerpinB2, also known as plasminogen activator inhibitor type 2 (PAI-2), is widely described as an inhibitor of the extracellular urokinase plasminogen activator (uPA), and to a lesser extent, tissue plasminogen activator (tPA) [1]. SerpinB2 is induced by a range of pro-inflammatory stimuli and viral, bacterial and parasitic infections [1], [2]. SerpinB2 is expressed by monocytes/macrophages, dendritic cells, neutrophils and eosinophils, and a range of non-haematopoietic cells. In activated monocytes/macrophages SerpinB2 is often one of the most up-regulated genes and can represent >0.25% of total cellular protein [1]. Despite ≈950 publications on SerpinB2/PAI-2 in PubMed and the association of SerpinB2 with a range of activities *in vitro*, a consensus view on the physiological function of SerpinB2 has yet to emerge [1]. Using SerpinB2^−/−^ mice we recently obtained evidence that at least one function of SerpinB2 is sculpting of the adaptive immune response, with SerpinB2 expression associated with suppression of certain Th1 responses and/or promotion of certain Th2 responses [3], [4]. These observations are consistent with the association of dysregulated SerpinB2 expression or SerpinB2 polymorphisms with Th1/Th2 perturbations in several human inflammatory diseases [1].

Acute HIV-1 and SIV infections are associated with the widespread induction of pro-inflammatory cytokines, including TNF [5]. Since inflammation generally, and TNF in particular, are well known to induce SerpinB2 expression [1], [6], one might predict that these primate lentiviral infections would up-regulate SerpinB2 expression. *In vitro* experiments have reported induction of SerpinB2 (i) in macrophages by gp120 from M-tropic HIV [7], (ii) in monocyte-derived dendritic cells by HIV infection [8] and (iii) in peripheral blood mononuclear cells (PBMCs) stimulated with baculovirus-expressed HIV Pr55gag virus-like particles [9]. SerpinB2 has generally not been identified as being regulated during lentiviral infections *in vivo* [10], perhaps because (i) SerpinB2 fell outside the focus of the study, (ii) cells were used that may not express SerpinB2 (eg T cells) [11], or (iii) array platforms were used that did not contain SerpinB2 [12]. *In vitro* experiments using cell lines stably expressing SerpinB2 suggested SerpinB2 expression might facilitate HIV replication [7]. However, this work was largely based on SerpinB2-expressing cell lines generated by transfection and selection, a process that can result in clonal bias [1], [13]. Nevertheless, preferential replication of SIV in monkey monocytes expressing SerpinB2 *in vivo* was observed in a microarray study [14]. Global genomic or RNAi screens have not identified SerpinB2 as an important host protein for HIV replication [15], [16], [17]. However, the cell lines used do not usually express significant levels of SerpinB2; for instance, SerpinB2 expression is ordinarily undetectable in HeLa [18] and HEK293 cells [19]. Another microarray study showed that effective vaccination was associated with higher SerpinB2 expression (when compared with unvaccinated controls) in PBMC following SIV challenge [20], perhaps supporting the view that SerpinB2 has a role in immunity [1].

To obtain a clearer understanding of whether SerpinB2 is induced during lentivirus infections *in vivo*, we analyzed SerpinB2 mRNA and protein expression in serial PBMC samples obtained from pigtail macaques infected with 2 primate lentiviruses; (i) SIV_mac251_, which utilizes the CCR5 co-receptor to enter cells and therefore readily infects macrophages as well as memory CD4 T cells [21], or (ii) the highly pathogenic SHIV_mn229_, which utilizes the CXCR4 co-receptor to enter cells and therefore primarily infects naïve CD4 T cells [22]. We found SerpinB2 was induced in SIV infections, and we therefore investigated the potential role of SerpinB2 in lentiviral infections *in vivo* by infecting SerpinB2^−/−^ and SerpinB2^+/+^ littermate control mice [4] with EcoHIV. EcoHIV is a chimeric human immunodeficiency virus type 1 (HIV-1), in which gp120 has been replaced with gp80 from ecotropic murine leukemia virus [23]. Taken together with analysis of published microarray data from human HIV-1 infections, the data presented herein supports the view that SerpinB2 is induced during SIV and HIV infections, and that SerpinB2 modulates anti-lentiviral Th1/Th2 responses.

## Materials and Methods

### Monkey Infections and Monitoring

SIV_mac251_ and SHIV_mn229_ infection of pigtail macaques (*Macaca nemestrina*) and gathering of clinical and immunological data were undertaken as described [21], [22]. All monkey experiments were approved by the University of Melbourne animal ethics committee and adhered to the National Health and Medical Research Council (Australia) code of practice for the care and use of animals. Viral loads were determined by quantitative real-time reverse transcriptase PCR (qRT-PCR) as described [24]. SIV-specific T cell responses were assessed by intracellular cytokine staining for IFNγ expression using overlapping 15 mer peptide sets spanning SIVmac239 gag (NIH AIDS Reagent repository) as described [21], [24]. The percentage of CD4^+^ cells in the CD3^+^ population from PBMC was determined by FACS as described [24].

### qRT-PCR of Monkey and Mouse Samples

Cryogenically stored PBMCs (4–7×10^6^) from SIV_mac251_ (Macaque animal numbers 3C7D, 3117, 5612, 5831 6115, 6158, 6288) [21] and SHIV_mn229_ (Macaque animal numbers 1253, 1281, 4194, 4265, 4529 and H3) [22] infected pigtail macaques were rapidly thawed in a 37°C water bath, diluted in 10 ml of ice cold RPMI1640 supplemented with 10% fetal calf serum and were pelleted by centrifugation at 300×g for 5 min at 4°C. Cell pellets were resuspended in 1 ml of TRIzol reagent (Invitrogen) and RNA extracted according to manufacturer’s instructions. RNA quality and quantity was assessed by NanoDrop ND-1000 spectrophotometer (Thermo Scientific, Waltham, MA, USA) and gel electrophoresis. cDNA was synthesized as described previously [4]. qRT-PCR was undertaken as described [4] using the following primers; SerpinB2 forward GACCAGATGGCCAAGGTGC, SerpinB2 reverse GAGAGAGCGGAAGGATGAATGG. SerpinB2 mRNA levels were normalized to RPL13A mRNA levels; primers were RPL13A forward CATCGTGGCTAAACAGGTACTG, RPL13A reverse CGCACGACCTTGAGGGCAGC.

Murine spleens were homogenized in TRIzol using 5 mm steel ball bearings and a TissueLyser (Qiagen, Venlo, NL) and peritoneal exudates cells (PECs) were lysed directly in TRIzol reagent. All RNA samples were treated with DNAse I (New England Biolabs, Ipswich, MA, USA) as per manufacturer’s instructions prior to cDNA synthesis and qRT-PCR as described above. The primers used were MLV env forward TAGGGCCAAACCCCGTTCTG, MLV env reverse GCCGGTGGAAGTTGGGTAGG. MLV env RNA levels were normalized to RPL13A mRNA levels; RPL13A forward GAGGTCGGGTGGAAGTACCA, RPL13A reverse TGCATCTTGGCCTTTTCCTT.

### SerpinB2 Protein Quantitation

Following RNA extraction from the aqueous TRIzol fraction (see above), protein was extracted from the interphase and phenol TRIzol fractions as per manufacturer’s instructions (Invitrogen). Protein pellets were solubilised in 1% SDS and heated at 50°C and total protein concentrations were determined by BCA protein assay (Pierce, Rockford, IL, USA). SerpinB2 protein levels were determined by IMUBIND® PAI-2 ELISA (American Diagnostica, Pfungstadt, Germany). Protein samples were diluted 1∶20 in ELISA sample buffer (American Diagnostica) and the ELISA was performed as per manufacturer’s instructions.

### EcoHIV

pEcoHIV was kind gift from Drs D Volsky and MJ Potash (St. Luke's-Roosevelt Hospital, Columbia University Medical Center, NY, USA). EcoHIV (NL4.3) was generated as described previously [23]. Briefly, EcoHIV plasmid (50 µg) was diluted to 500 µl in 2.5 mM HEPES buffer (pH 7.3), 1 ml of 0.5 M CaCl_2_ was then added, followed by 1 ml of 2× HEPES buffered saline (pH 7.05) added drop-wise whilst vortexing. The transfection mixture was incubated for 20 min at room temperature before being added drop-wise to a T150 flask of 80% confluent 293T cells. Sixteen hours post-transfection, cells were washed twice with 20 ml of PBS, and fresh culture medium added. Sixty-four and 88 h post-transfection, supernatants were collected and centrifuged at 1200×g for 7 min at 4°C, and the supernatants filtered through a 0.45 µm filter to remove cellular debris. EcoHIV was concentrated by centrifugation of the filtrate at 3800×g for 20–30 min in a 100,000 molecular weight cut-off Amicon Ultra centrifugal filter (Millipore, Billerica, MA, USA). EcoHIV stocks were analysed by HIV-1 p24 ELISA Kit (Zeptometrix Corporation, Buffalo, NY, USA) according to manufacturer’s instructions to determine the concentration of p24 protein.

### Mice and EcoHIV Infection and Monitoring

SerpinB2^−/−^ and SerpinB2^+/+^ littermate control colonies on a C57BL/6 background were established as described [4]. Mice (6–12 weeks old females) were infected by intra-peritoneal injection of 2.5 µg p24 EcoHIV in 1 ml of DMEM. At the indicated time points mice were euthanised by CO_2_ asphyxiation and splenocytes, PECs and serum harvested. All mouse experiments were approved by the QIMR animal ethics committee and adhered to the National Health and Medical Research Council (Australia) code of practice for the care and use of animals.

### Anti-gag Antibody ELISA

MaxiSorp Nunc-Immuno 96 well plates (Nunc, Roskilde, DK, USA) were coated with 80 ng/well (in 40 µl) HIV-1 NL4-3 *gag* protein (ProspecBio, Israel) overnight at 4°C. Plates were then blocked with 200 µl of 5% skim milk in PBS for 1 hour at room temperature, and washed three times in PBS/0.01%Tween20 (PBS/T). Serum (60 µl/well) was serially diluted (1∶2) in 1% skim milk/PBS/T and added to the wells at room temperature for 2 h. After washing, 70 µl of biotin-conjugated rat anti-mouse IgG1 (1∶1500) (A85-1, BD Bioscience, Franklin Lakes, NJ, USA) or biotin-conjugated rat anti-mouse IgG2c (1∶800) (R19-15) diluted in PBS/T was added to each well, and the plate was incubated at room temperature for 2 hours. After washing with PBS/T, 100 µl of streptavidin-HRP (BioSource International, Camarillo, CA) in PBS/T (1∶10,000) was added to each well and incubated for 45 min. The plate was developed by adding 100 µl of ABTS:H_2_O_2_ (1000∶1 ratio) (Sigma-Aldrich) and absorbance was read at 405 nm.

### Statistics

Statistical analysis was performed using SPSS for Windows (version 19; SPSS, Chicago, IL, USA). For comparison of two samples, the t test was used when the difference in the variances was <4 and skewness was >−2 and kurtosis was <2; otherwise, the non parametric Mann-Whitney U test was used. Correlations used the non-parametritic Spearman's rank correlation test, which provides a p value and a Spearman rank correlation coefficient (rho), which ranges from −1 (perfect negative correlation) to +1 (perfect positive correlation) with 0 denoting no correlation.

## Results

### SerpinB2 Expression is Increased after SIV and Decreased after SHIV Infection

**Figure 1:**
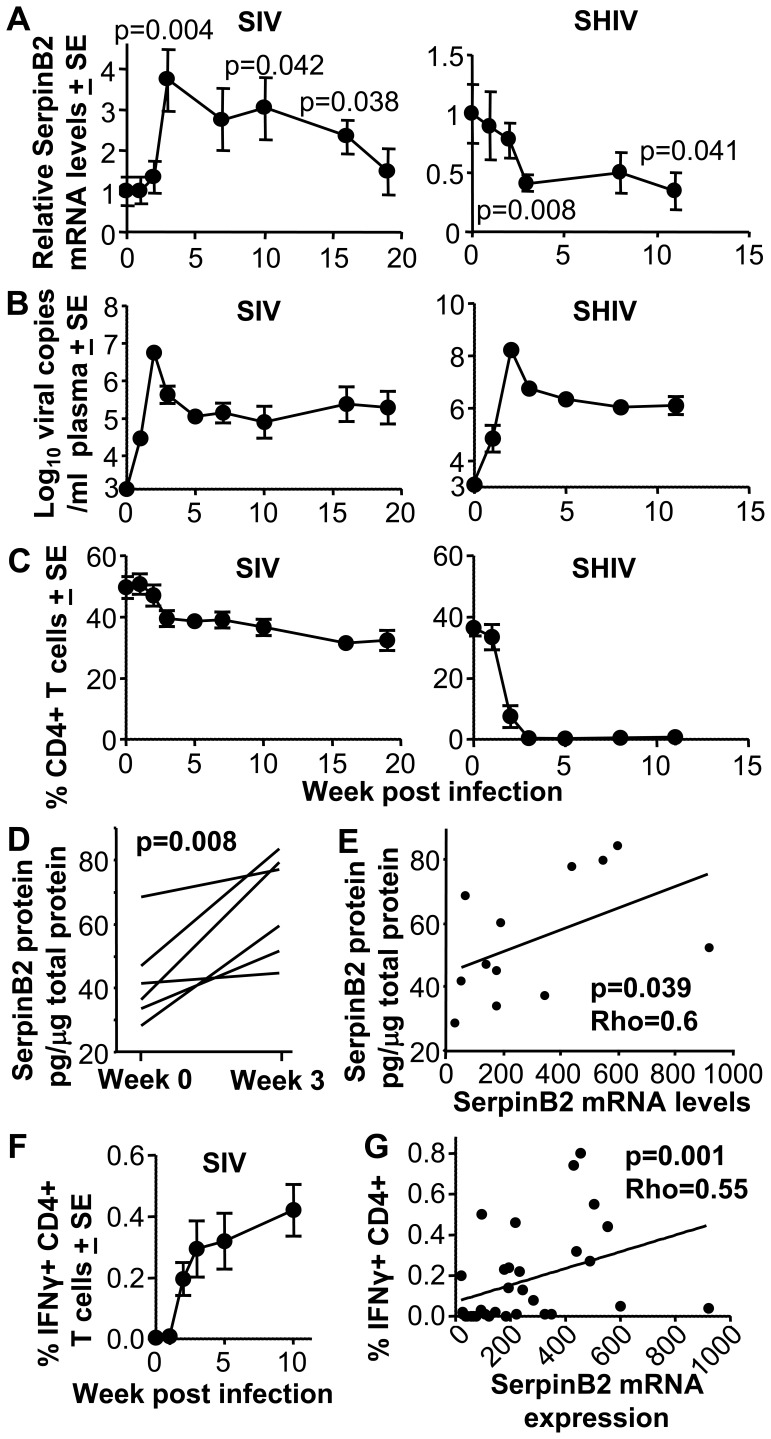
SIV and SHIV infection in monkeys. (A) SerpinB2 mRNA expression levels in PBMCs after SIV_mac251_ and SHIV_mn229_ infection of pigtail macaques. Expression levels were determined by qRT-PCR and normalised against RPL13A mRNA levels. Levels are shown relative to the mean levels seen in monkeys prior to infection (day 0), (n = 4–7 samples per time point). Statistics by Mann Whitney U test indicating significant differences relative to day 0. (B) Plasma viral loads for the same samples described in A expressed as log_10_ RNA copies per ml. The detection limit was 3.2 log_10_ viral copies per ml. (C) CD4 T cell percentages for the same samples described in A, expressed as the percentage of CD4^+^ cells in the CD3^+^ PBMC population. (D) SerpinB2 protein levels in PBMC samples from the SIV infected monkey described above taken at week 0 & 3 and determined by quantitative ELISA. Each line represents one monkey. Statistics by paired t test. (E) Spearman correlation of SerpinB2 protein levels (shown in D) and SerpinB2 mRNA levels (described in A, SIV) from the same PBMC samples. (F) Percentage of IFNγ secreting CD4^+^ T cells in SIV infected monkeys after stimulation with a gag peptide pool using the same PBMC samples described in A (except for day 15–20 samples). Data is expressed as the percentage of IFNγ^+^/CD4^+^ T cells within the CD4^+^/CD3^+^ population. (G) Spearman correlation of (i) percentage of IFNγ secreting CD4^+^ T cells after gag peptide stimulation (shown in F) and (ii) the SerpinB2 mRNA expression levels normalised against RPL13A mRNA (used to generate A, SIV), derived from the same PBMC samples.

SerpinB2 expression levels during acute lentiviral infections *in vivo* have not previously been investigated. We thus quantitated SerpinB2 mRNA expression levels using qRT-PCR in serial PBMC samples from pigtail macaques following infection with either SIV_mac251_ [21] or the pathogenic SHIV_mn229_ [22]. Three weeks after SIV infection SerpinB2 mRNA levels had increased by a mean 3.7 fold (range 1.4–4.7, n = 7 per group, p = 0.004), and remained significantly elevated until week 16 (mean 2.3 fold, p = 0.03) (Fig. 1A, SIV). The viral load in these monkeys peaked at week 2 post infection, one week prior to the peak SerpinB2 induction (Fig. 1B, SIV). CD4 T cell counts declined slowly over the study period of 20 weeks (Fig. 1C, SIV), as observed previously [21], [24]. In contrast, infection with the CXCR4-tropic SHIV_mn229_ resulted in a reduction in SerpinB2 mRNA levels in PBMCs; levels had fallen significantly by week 3 and remained depressed until week 11 (Fig. 1A, SHIV, p = 0.008 and 0.04, respectively). SHIV infection also resulted in a peak viral load at week 2 (Fig. 1B, SHIV), and was characterized by rapid T cell depletion, with all peripheral CD4 T cells essentially lost by week 3 (Fig 1C, SHIV). The dramatic and near complete loss of peripheral CD4 T cells is typical of infection of macaques with CXCR4-utilizing SHIV strains [22].

To confirm that the significant increase in SerpinB2 mRNA levels seen at 3 weeks post SIV infection (Fig. 1A) reflected an increase in SerpinB2 protein levels, total protein from the same samples used to determine mRNA levels were assayed for SerpinB2 protein levels using a quantative ELISA. A significant increase in SerpinB2 protein levels was observed between week 0 and week 3 (p = 0.008 paired t test). SerpinB2 protein levels in PBMC samples increased by a mean of 63% (SD 47% and range 8–115%). Although post-transcriptional regulation of SerpinB2 expression has been well documented [25], a significant correlation between SerpinB2 protein and mRNA levels was observed (Fig. 1E).

The percentage of CD4 and CD8 T cells making IFNγ (as determined by intracellular cytokine staining of cells stimulated with overlapping peptides covering gag) following SIV infection increased sharply until week 2–3 post infection before leveling out (Fig. 1F), consistent with previous analyses in this model [26]. Similar assays for SHIV-infected animals showed negligible T cell immunity due to the loss of T cells in this rapidly pathogenic infection (data not shown, see also [26]). Using data for all SIV-infected monkeys at all time points, no significant correlation between viral loads, CD4 counts, percentage of CD8 T cells making IFNγ, and SerpinB2 mRNA expression levels emerged (data not shown). A significant correlation did emerge between the percentage of antigen-specific CD4 T cells (as measured by intracellular IFNγ staining) and the levels of SerpinB2 mRNA (Fig. 1G). SIV-specific CD4 cells are known to make TNF [27], [28], [29] and TNF is well known to induce SerpinB2 [6].

### Expression of SerpinB2 mRNA after EcoHIV Infection

**Figure 2:**
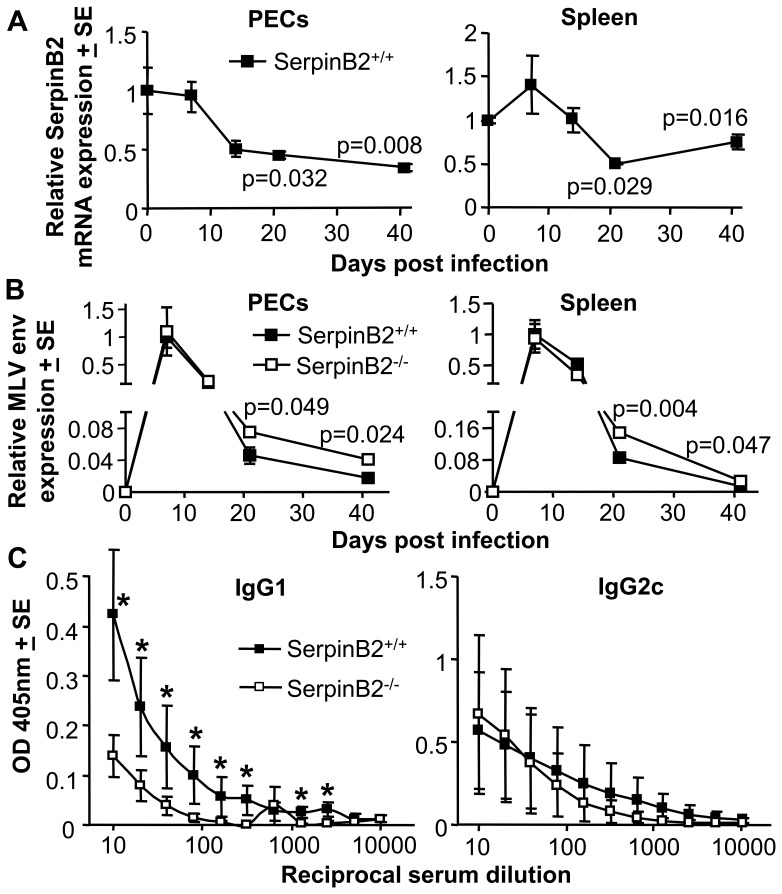
EcoHIV infection of SerpinB2^+/+^ and SerpinB2^−/−^ mice. (A) SerpinB2 mRNA expression levels in peritoneal exudate cells (PECs) and spleen after EcoHIV infection of SerpinB2^+/+^ mice. Expression levels were determined by qRT-PCR and normalised against RPL13A mRNA levels. Levels are shown relative to the mean levels seen in mice prior to infection (day 0), (n = 4–5 samples per time point). Statistics by Mann Whitney U test indicating significant differences relative to day 0. (B) EcoHIV RNA levels in PECs and spleen after EcoHIV infection of SerpinB2^+/+^ and SerpinB2^−/−^ mice. Levels were determined by qRT-PCR using MLV env primers and normalised against RPL13A mRNA levels. Levels are shown relative to the mean levels seen in SerpinB2^+/+^ mice on day 7 (peak viraemia), (n = 4–5 samples per time point). Statistics as for A. (C) HIV gag-specific IgG1 and IgG2c serum antibody responses 21 days after EcoHIV infection of SerpinB2^+/+^ and SerpinB2^−/−^ mice. *indicates statistical difference (p<0.028 ) by Mann Whitney U tests between OD values for SerpinB2^+/+^ and SerpinB2^−/−^ mice (n = 5 per group).

To determine whether SerpinB2 is induced in mice after EcoHIV infection, SerpinB2^+/+^ mice were infected by intra-peritoneal injection with EcoHIV. The levels of SerpinB2 mRNA in spleen and peritoneal exudate cells (PECs) were measured over time by qRT-PCR. The SerpinB2 mRNA levels in EcoHIV infected mice decreased significantly by ≈50% on days 21 and 41 post infection in both PECs (p = 0.032 and 0.008, respectively) and spleen (p = 0.029 and 0.016, respectively) (Fig. 2A). This occurred despite an influx of macrophages into the peritoneum (see below).

EcoHIV infects macrophages and does not result in CD4 T cell depletion [23]. To understand why EcoHIV infection failed to induce SerpinB2 expression, cytokine mRNA levels in spleen and PECs were analysed by qRT-PCR at different times post-infection. No significant induction of IFNγ or TNF was apparent in PECs or spleen (Figure S1 in File S1); an observation consistent with the modest antigen-specific IFNγ T cell responses seen in this model [30]. In contrast, significant induction of IL-6 (∼25 fold) and IL-4 mRNA (6–10 fold) was observed in PECs (Figure S1 in File S1). IL-4 (a Th2 cytokine) has been reported to down-regulate SerpinB2 expression [6]. We were also unable to observe induction of SerpinB2 in mouse peritoneal macrophages after EcoHIV infection *in vitro* (data not shown). This contrasts with M-tropic HIV infection of human PBMCs *in vitro,* where SerpinB2 mRNA induction was observed [7].

### EcoHIV Replication in SerpinB2^+/+^ and SerpinB2^−/−^ Mice

To determine whether SerpinB2 might affect EcoHIV replication *in vivo*, SerpinB2^+/+^ and SerpinB2^−/−^ mice were infected with EcoHIV, and EcoHIV mRNA levels were measured by qRT-PCR using primers that detect all MLV env RNA species. In both PECs and spleens of EcoHIV infected mice, viral mRNA levels peaked at day 7 post-infection and progressively declined until day 41 (Fig. 2B). Although no differences in viral mRNA levels were observed in PECs or spleen from SerpinB2^−/−^ and SerpinB2^+/+^ mice at day 7 and 14, significantly higher (≈2 fold) viral mRNA levels were detected in SerpinB2^−/−^ PECs at day 21 (p = 0.049) and day 41 (p = 0.024) when compared with SerpinB2^+/+^ PECs (Fig. 2B, PECs). Higher EcoHIV mRNA levels (≈2 fold) were also observed in SerpinB2^−/−^ spleens at day 21 (p = 0.004) and day 41 (p = 0.047) when compared with spleens from SerpinB2^+/+^ mice (Fig. 2B). The significant difference in splenic EcoHIV mRNA levels at day 21 was confirmed using different primers that only detect the 4 kb singly spliced sub-genomic env-1 RNA (Figure S2 in File S1).

We were unable to see any difference in EcoHIV replication in SerpinB2^−/−^ and SerpinB2^+/+^ macrophages *in vitro* by qRT-PCR (Figure S3 in File S1), suggesting that in this system (in contrast to [7]), SerpinB2 expression by an infected cell does not influence viral replication in that cell.

### Decreased IgG1 Responses in EcoHIV Infected SerpinB2^−/−^ Mice

We have previously shown that SerpinB2^−/−^ mice show increased levels of IgG2c responses (a Th1-associated antibody isotype) after vaccination with antigen in Freunds complete adjuvant [1] (a predominantly Th1 immunogen) and reduced levels of IgG1 responses (a Th2-associated antibody isotype) after vaccination with schistosome soluble egg antigen (a predominantly Th2 immunogen) [3]. Analysis of anti-gag antibody responses in EcoHIV-infected mice showed that SerpinB2^−/−^ mice produced significantly lower levels of IgG1 responses, with no significant differences in IgG2c responses (Fig. 2C). The experiment was repeated with similar results (Figure S4 in File S1). Naïve uninfected mice did not show any significant anti-gag antibody responses (data not shown). Thus, as described for schistosome soluble egg antigen vaccination [3], SerpinB2 expression appeared to be associated with increased Th2 responses following EcoHIV infection.

We previously reported changes in TNF, IFNγ, IL-4 and/or IL-6 levels in SerpinB2^−/−^ mice [3], [4]; however, no significant and consistent differences in these cytokines (as measured by qRT-PCR) were apparent between EcoHIV-infected SerpinB2^−/−^ and SerpinB2^+/+^ mice (Figure S1 in File S1). Conceivably, other cytokines are involved and/or significant Th1/Th2 cytokine differences are present in other sites or cell types.

### Cell Migration was Unchanged in EcoHIV-infected SerpinB2^−/−^ Mice

Several studies have suggested that SerpinB2 inhibits uPA-dependent cellular migration [1], although no change in lymph node composition was seen in SerpinB2^−/−^ mice after immunisation with Freunds complete adjuvant [4]. The intra-peritoneal EcoHIV infection did result in a progressive increase in the total number of cells in the peritoneum; however, no significant differences between SerpinB2^−/−^ and SerpinB2^+/+^ mice were observed (Figure S5 in File S1). The levels of macrophage-specific F4/80 mRNA also increased in PECs post infection, but again there was no significant difference between the two mouse strains (Figure S6 in File S1).

### Analysis of Published Microarray Data from Human HIV-1 Infected Individuals

**Figure 3:**
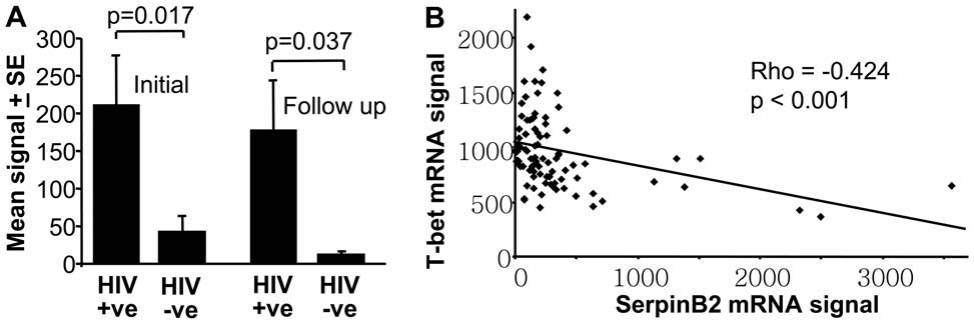
Analysis of published microarray data from human HIV-1-infections *in vivo*. (A) Mean SerpinB2 mRNA levels from published microarray analysis of monocytes isolated from chronic HIV viraemic patients not on therapy [31], [32]. The initial analysis involved n = 12 HIV positive (+ve) and n = 13 HIV negative (–ve) patients, with a follow-up study involving some of the same patients, n = 4 HIV+ve and n = 4 HIV–ve. The expression data was obtained from NCBI GEO database (GSE14542). Statistics by Mann Whitney U test. (B) Negative correlation between SerpinB2 and T-bet mRNA levels using published [34] microarray data from PBMCs isolated from HIV-1 (n = 75) and control (n = 12) patients. Data was obtained from the NCBI GEO database (GDS1449). Statistical analysis was performed using Spearman’s rank correlation test; both p value and Spearman rank correlation coefficient (rho) are shown.

To determine whether any evidence for SerpinB2 induction and Th1/Th2 modulation could be found for HIV-1 infected humans, we searched NCBI GEO for informative microarray data sets. In one study of chronic HIV viraemic patients not on therapy, monocyte SerpinB2 mRNA levels were found to be significantly higher when compared with uninfected controls, both in an initial analysis and a follow up study [31], [32] (Fig. 3A). In another study, significantly higher levels of SerpinB2 mRNA were reported for CD4 T cells from HIV infected individuals and elite controllers when compared to uninfected controls [33] (Figure S7 in File S1). However, in this study samples with high SerpinB2 mRNA levels often also had higher levels of CD14 or CD64 mRNA (data not shown herein), suggesting that contaminating monocytes may have contributed to SerpinB2 signals. To date, SerpinB2 expression by primary T cells has not been formally demonstrated.

Analysis of data from a microarray study of PBMCs from HIV-1 infected and uninfected control individuals [34], did not show a significant difference in SerpinB2 levels between HIV-1 infected and controls, perhaps because most of the HIV-1 infected individuals were on treatment. However, the analysis did show a highly significant (p<0.001) negative correlation between SerpinB2 and T-bet mRNA levels (Fig. 3B). T-bet is a transcription factor that drives differentiation of Th1 T cells. A slight, but significant (p = 0.022), positive correlation between SerpinB2 and IL-4 mRNA expression levels was also evident (Figure S8 in File S1). Thus higher SerpinB2 expression was again associated with lower Th1 (and increased Th2) responses.

These analyses of published microarray studies thus provide evidence that during HIV-1 infections in humans, SerpinB2 is induced in monocytes and that SerpinB2 expression correlates with modulation of Th1/Th2 responses.

## Discussion

The induction of SerpinB2 mRNA in PBMCs after infection of monkeys with the M-tropic SIV_mac251_ is consistent with microarray studies showing significantly increased SerpinB2 mRNA in circulating monocytes from HIV-1 infected patients and supports the general concept that monocyte/macrophage SerpinB2 is often induced during infection and inflammation [1]. One should also perhaps note that analyzing circulating cells may under-represent SerpinB2 induction during SIV/HIV infections, as viral replication and inflammatory cytokine levels (and thus SerpinB2 induction) may be higher in secondary lymphoid organs and/or the gut [35], [36].

Although the physiological role of SerpinB2 remains elusive, the evidence from the EcoHIV mouse model (Fig. 2C) and the analysis of published human microarray data (Fig. 3B) presented herein supports the emerging concept that at least one of the roles of monocyte/macrophage SerpinB2 is suppression of Th1 and/or promotion of Th2 responses [1]. Such modulation by SerpinB2 has now been shown after vaccination [4], after parasite antigen vaccination [3] and (herein) after lentiviral infections. SerpinB2^−/−^ mice infected with EcoHIV produced significantly lower gag-specific IgG1 responses (Fig. 2C); a Th2 modulation similar to that seen in SerpinB2^−/−^ mice vaccinated with schistosome egg antigen [3]. The clear IL-4 response seen after EcoHIV infection (Fig. S1), suggests EcoHIV infection (like schistosome egg antigen vaccination [3]) induces a Th2-biased response. Thus in these two very different Th2-biased challenge models, SerpinB2 expression appears to promote Th2 responses. The human microarray analysis (Fig. 3B) suggests that during HIV-1 infection in humans, higher SerpinB2 mRNA levels correlate with suppression of Th1 (T-bet) responses; a Th1 modulation similar to that seen in SerpinB2^−/−^ mice after vaccination with a Th1-promoting vaccine [4].

The effects of SerpinB2 deficiency on viral load were restricted to later time points (days 21 and 41) (Fig. 2B), with SerpinB2^−/−^ mice showing 40–50% higher viral RNA levels at these times. One might speculate (based on [37]) that these later differences are related to the lower anti-viral IgG1 antibody levels that develop in SerpinB2^−/−^ mice. This contention is also supported by the negative correlation between anti-EcoHIV IgG1 levels and EcoHIV RNA levels, although this did not reach significance (Figure S9 in File S1). Importantly, the EcoHIV infection model both *in vivo* (Fig. 2B) and *in vitro* (Figure S3 in File S1) suggests that SerpinB2 expression by the infected cell does not affect lentiviral replication directly, as was suggested by previous studies [7]. Instead, SerpinB2 may affect viral clearance via modulation of adaptive immune responses.

The lack of SerpinB2 mRNA induction following EcoHIV infection may be due to (i) acute induction of IL-4, which can down-regulate SerpinB2 expression [6] and/or (ii) the failure of MLV env to stimulate appropriate signalling in monocytes/macrophages. These two features of EcoHIV infections differ from human HIV infections, where Th1 cytokines usually dominate during the acute phase [38], and gp120 and infection can induce SerpinB2 expression in monocytes/macrophages *in vitro* [7]. The CXCR4-tropic SHIV-infection in pigtail macaques may also represent a less relevant model for most acute HIV infections in humans, where natural transmission is largely restricted to CCR5-tropic viruses [39]. Why the SHIV infection failed to induce SerpinB2 expression remains unclear, but may be due to either: (i) different monocytes/macrophage targeting and/or stimulation by this CXCR4-tropic virus or the gp120 from this virus [40], [41]; or (ii) less microbial translocation induced by SHIV [42] as compared with SIV [43], with lipopolysaccharide well known to induce macrophage SerpinB2 expression [1]; or (iii) less TNF production (possibly by CD4 T cells - Fig. 1G); with TNF detectable in serum of SIV_mac251_ infected monkeys [44], but not in certain SHIV infections [45]; or (iv) conceivably, loss of activated/infected CD4 T cells themselves. Microarray studies have suggested that activated and infected CD4 T cells up-regulate SerpinB2 mRNA [33], [46], although low levels of monocyte contamination could account for these results (Figure S7 in File S1). To our knowledge there are no reports showing that primary T cells make SerpinB2 protein.

Both SIV and HIV infections appear to induce SerpinB2 expression *in vivo*. SerpinB2 has been shown to modulate Th1/Th2 responses in the EcoHIV mouse model (Fig. 2C) and two other mouse models [3], [4], with correlative evidence suggesting this may also be the case in human HIV-1 infections *in vivo* (Fig. 3B) and several other human inflammatory diseases [1]. There may thus be value in understanding the relationship between immune dysregulation and SerpinB2 expression in HIV infections *in vivo* [38]. The role of SerpinB2 polymorphisms may also warrant investigation as they have been linked with both lupus [47] and coronary heart disease [48], conditions also modified by HIV infection [49], [50].

## Supporting Information

File S1 Figure S1. Cytokine mRNA levels in spleen and PECs as measured by qRT-PCR after EcoHIV infection. Figure S2. qRT-PCR of 4 kb singly spliced sub-genomic env-1 RNA in spleen 21 days after Eco HIV infection. Figure S3. qRT-PCR measuring EcoHIV infection levels in SerpinB2^−/−^ and SerpinB2^+/+^ macrophages *in vitro.* Figure S4. IgG1 responses from a repeat experiment to that described in Fig. 2C. Figure S5. Increase in PECs following EcoHIV infection. Figure S6. Increase in F4/80 mRNA levels in PECs 41 days after EcoHIV infection. Figure S7. Analysis of published microarray showing higher SerpinB2 mRNA levels in HIV infected verses control patients. Figure S8. Positive correlation between SerpinB2 and IL-4 mRNA levels in published microarray data from HIV infected and control patients. Figure S9. Negative correlation between anti-EcoHIV IgG1 levels and EcoHIV RNA levels.
(PDF)
