# Supplementary material for: Induction of SerpinB2 and Th1/Th2 Modulation by SerpinB2 during Lentiviral Infections In Vivo
Source: PLoS One. 2013 Feb 27;8(2):e57343. doi: 10.1371/journal.pone.0057343 (PMC3583835; doi:10.1371/journal.pone.0057343)
Supplement: File S1 — Figure S1. Cytokine mRNA levels in spleen and PECs as measured by qRT-PCR after EcoHIV infection. Figure S2. qRT-PCR of 4 kb singly spliced sub-genomic env-1 RNA in spleen 21 days after Eco HIV infection. Figure S3. qRT-PCR measuring EcoHIV infection levels in SerpinB2−/− and SerpinB2+/+ macrophages in vitro. Figure S4. IgG1 responses from a repeat experiment to that described in Fig. 2C. Figure S5. Increase in PECs following EcoHIV infection. Figure S6. Increase in F4/80 mRNA levels in PECs 41 days after EcoHIV infection. Figure S7. Analysis of published microarray showing higher SerpinB2 mRNA levels in HIV infected verses control patients. Figure S8. Positive correlation between SerpinB2 and IL-4 mRNA levels in published microarray data from HIV infected and control patients. Figure S9. Negative correlation between anti-EcoHIV IgG1 levels and EcoHIV RNA levels. (PDF) [file pone.0057343.s001.pdf]

Supporting Information

Figure S1. qRT-PCR measuring cytokine mRNA levels after EcoHIV infection of SerpinB2$^{-/-}$ and SerpinB2$^{+/+}$ mice (n=4/5 mice per group and time point). All cytokine mRNA levels were first normalized to RPL13A mRNA levels in the same sample, and are graphed relative to day 0 set at 1. Significant difference by Mann Whitney U test are shown for comparisons between the values at the indicated time and day 0 for the same mouse strain.

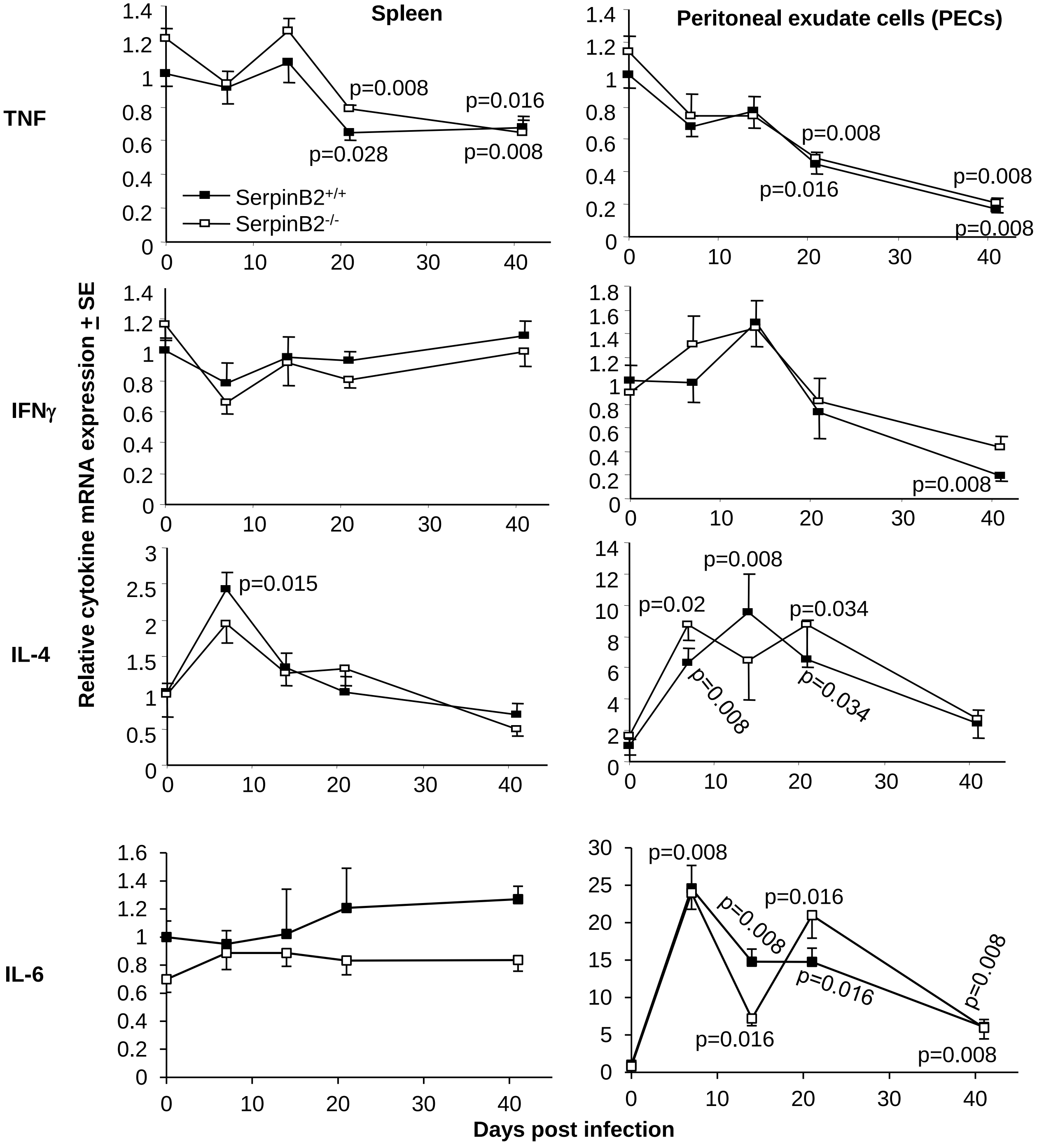

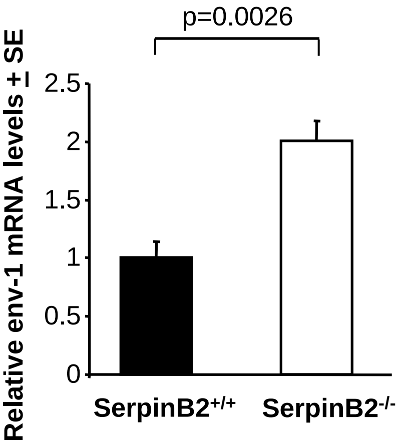

Figure S2. qRT-PCR of 4 kb singly spliced sub-genomic env-1 RNA . qRT-PCR of spleen day 21 post-infection with EcoHIV using env primers that detect the 4kb sub-genomic env-1 EcoHIV RNA. Primers were forward 5-GCGGCGACTGGAAGAAGC-3′ and reverse 5′-CTATGGACCACACAACTATTGC-3′. The forward primer spans the SD1/SA5 splice junction. The EcoHIV RNA mRNA levels were normalised to RPL13A mRNA levels and are presented relative to the mean levels seen in spleens from SerpinB2$^{+/+}$ mice. Statistics by t test, (n=4-5)

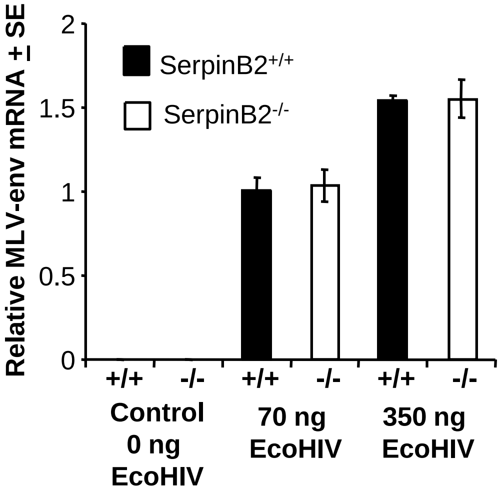

Figure S3. EcoHIV infection of SerpinB2$^{-/-}$ and SerpinB2$^{+/+}$ macrophages *in vitro*. Resident peritoneal macrophages (4 x 10^6 per well) were infected with EcoHIV *in vitro* with the indicated amount of p24 (as determined by ELISA analysis of EcoHIV viral stocks). Six days post-infection, MLV env mRNA levels were determined by qRT-PCR. Env mRNA levels were normalised to RPL13A mRNA levels, and are presented relative to the mean env mRNA levels seen in SerpinB2$^{+/+}$ macrophages after infection with 70 ng p24. Similar results were obtained when primers that detect the 4 kb sub-genomic env-1 RNA were used (data not shown). Resident peritoneal macrophages from SerpinB2$^{+/+}$ mice express high levels of SerpinB2 (Schroder et al., 2010).

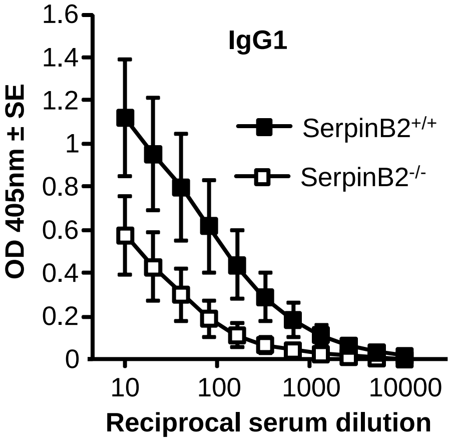

Figure S4. IgG1 responses. Repeat of the experiment described in Fig. 2C (n= 4 SerpinB2$^{+/+}$ and 3 SerpinB2$^{-/-}$ mice. Using a repeat measures ANOVA the two curves are significantly different ($p<0.001$). IgG2c responses again showed no significant differences (data not shown).

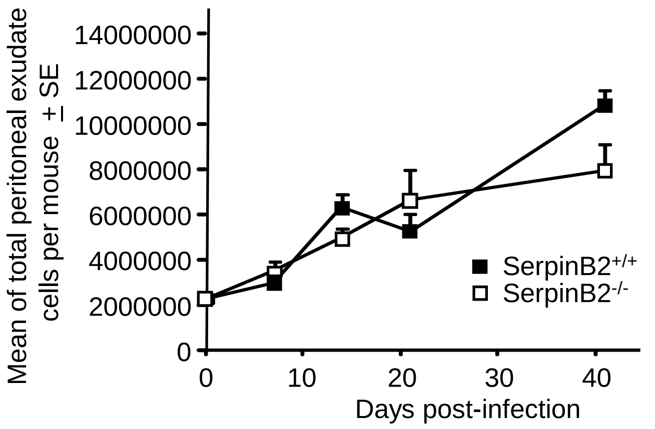

Figure S5. Increase in peritoneal exudate cells following EcoHIV infection. Peritoneal exudate cells (PECs) were harvested from uninfected (day 0) or infected mice at the indicated times (n=4 or 5 per group) by peritoneal lavage and counted using a haemocytometer.

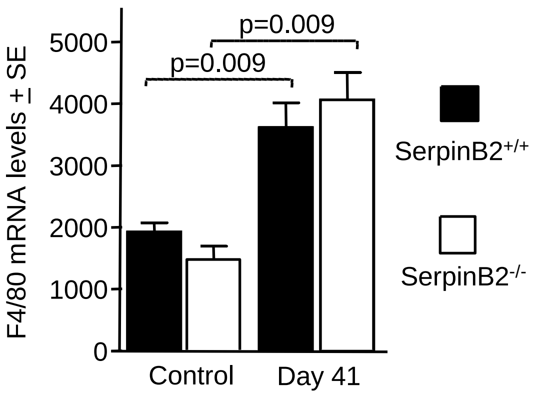

Figure S6. Macrophage F4/80 mRNA expression levels in peritoneal exudate cells. Levels of F4/80 mRNA levels in peritoneal exudate cells in uninfected (day 0) or EcoHIV infected mice day 41 post infection (n=4 or 5 per group) were determined by qRT PCR. Values were normalised to RPL13A mRNA levels. F4/80 primers were forward 5′-AGACTTGATACTCCAAAGTGAGC-3′ and reverse 5′-GAGACTTGTAGCTGCACTCTG-3′. Statistics by Mann Whitney U test. These results suggest that the number of macrophages in the PECs was not affected by SerpinB2 deficiency.

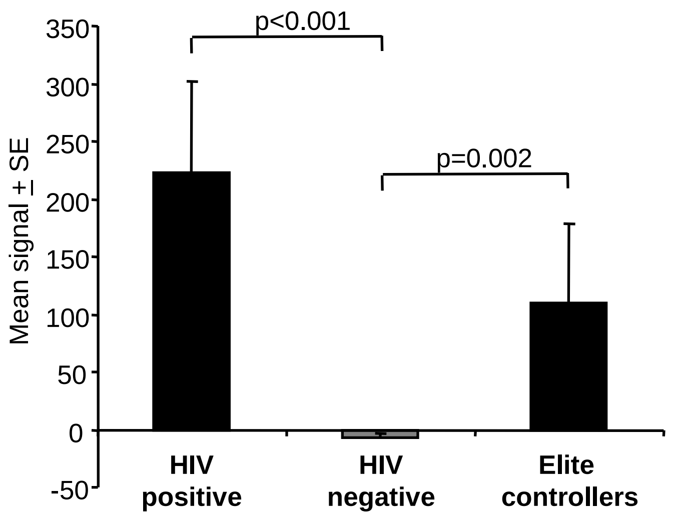

Figure S7. SerpinB2 levels in published HIV microarray studies. Mean SerpinB2 mRNA levels from microarray studies of isolated CD4 T cells from HIV-1 infected, HIV-negative and HIV-1 elite controllers (n=14, 9 and 12, respectively). Data obtained from NCBI GEO data base linked to (Vigneault et al. 2011). Samples with high SerpinB2 mRNA levels often also had higher levels of CD14 or CD64 mRNA (not shown), suggesting that contaminating monocytes may have contributed to SerpinB2 signals. Statistics by Mann Whitney U test.

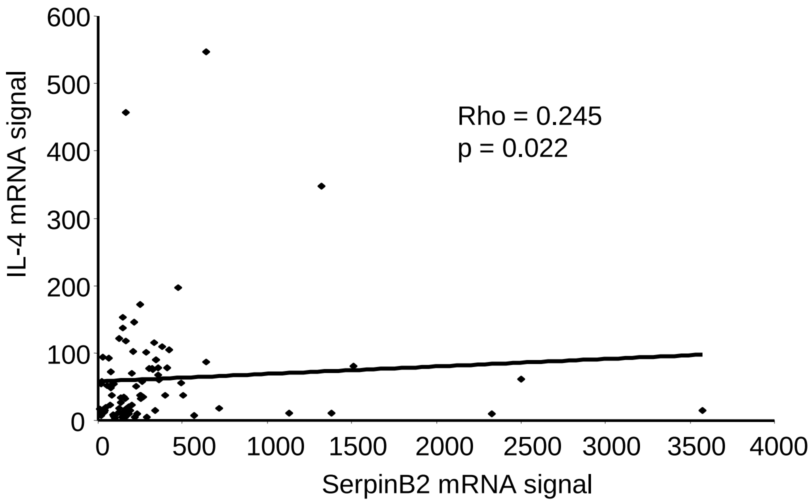

Figure S8. **Positive correlation between SerpinB2 and IL-4 mRNA levels.** Correlation between SerpinB2 and IL-4 mRNA levels using published (Ockenhouse et al., 2005) microarray data from PBMC isolated from HIV-1 (n=75) and control (n=12) patients. Data was obtained from NCBI GEO data base. Statistical analysis was performed using Spearman's rank correlation test; both p value and Spearman rank correlation coefficient (rho) are shown.

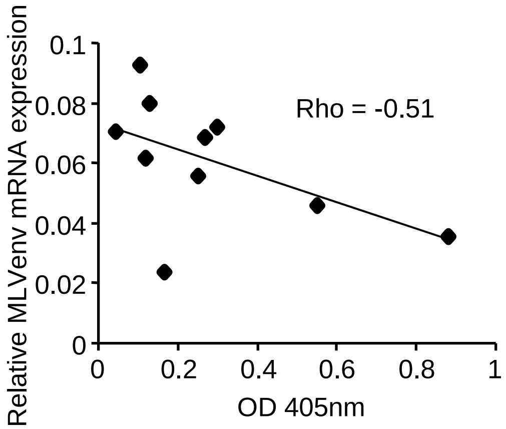

Figure S9. Negative correlation between anti-EcoHIV IgG1 levels and EcoHIV RNA levels. The OD values from Fig. 2C (IgG1, 1/10 dilution) representing the anti-gag antibody levels plotted against the relative day 21 PEC MLV env mRNA levels (from Fig. 2B) for each animal (n=5 SerpinB2$^{-/-}$ mice plus n=5 SerpinB2$^{+/+}$ mice). Spearman's rank correlation coefficient (rho) is provided indicating a negative correlation, although due to small sample size this did not reach significance.
